# Supplementary material for: Induction and inhibition of Drosophila X chromosome gene expression are both impeded by the dosage compensation complex
Source: G3 (Bethesda). 2022 Jul 6;12(9):jkac165. doi: 10.1093/g3journal/jkac165 (PMC9434221; doi:10.1093/g3journal/jkac165)
Supplement: jkac165_Supplementary_Figures [file jkac165_supplementary_figures.pdf]

SUPPLEMENTAL FIGURES

Induction and inhibition of *Drosophila* X chromosome gene expression are both impeded by the dosage compensation complex

Richard P. Meisel[1]*, Danial Asgari[1], Florencia Schlamp[2], Robert L. Unckless[3]

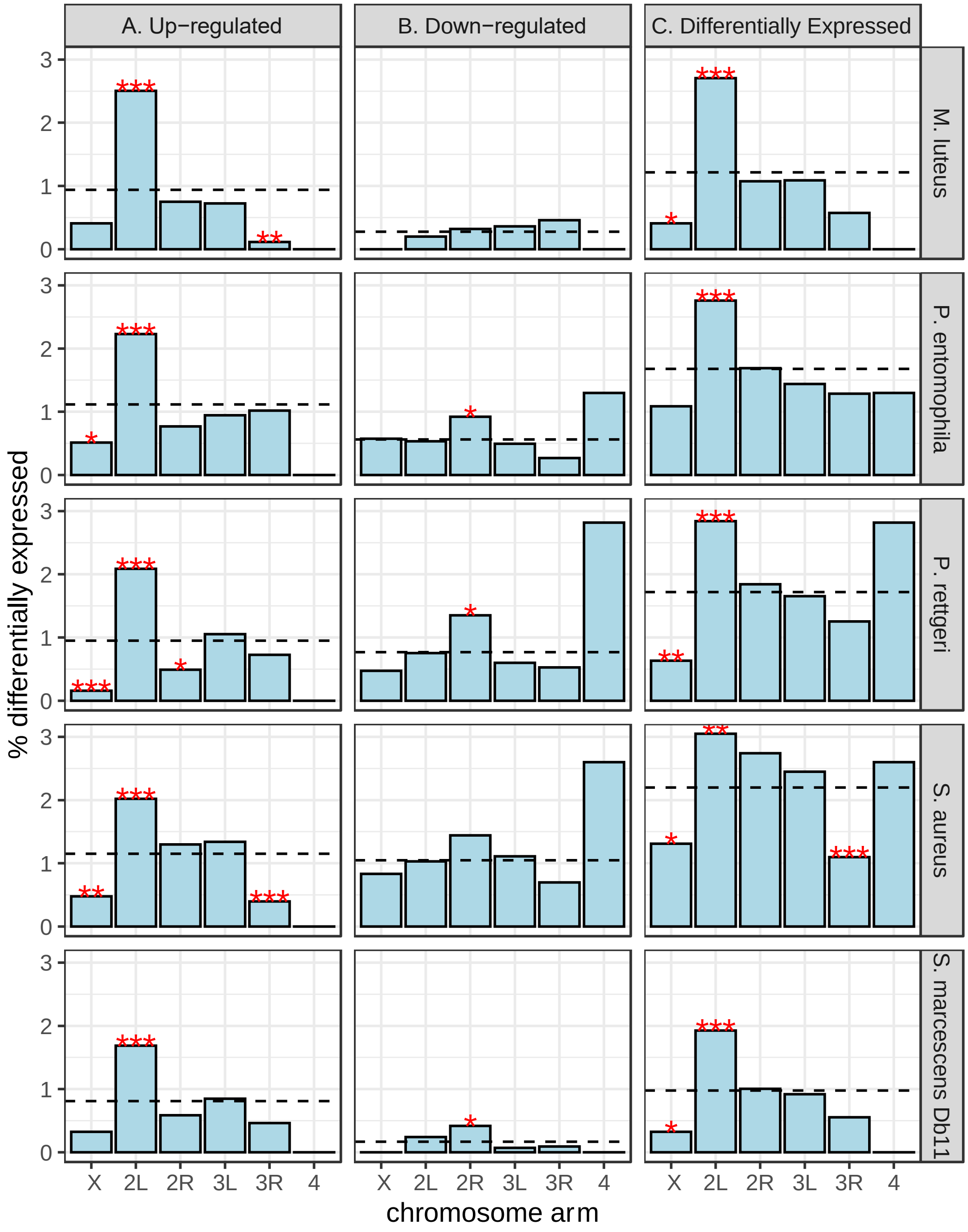

Supplemental Figure S1. The percent of genes on each chromosome arm that are differentially expressed following infection with one of five bacteria in male *D. melanogaster* is shown. Genes are either (A) up-regulated, (B) downregulated, or (C) differentially expressed (sum of up- and down-regulated). The percent of differentially expressed genes across the entire genome is shown as a dashed line. Asterisks indicate chromosomes where the percent of genes is significantly different from the rest of the genome (**P*<0.05, ***P*<0.005, or ****P*<0.0005 in Fisher's exact test).

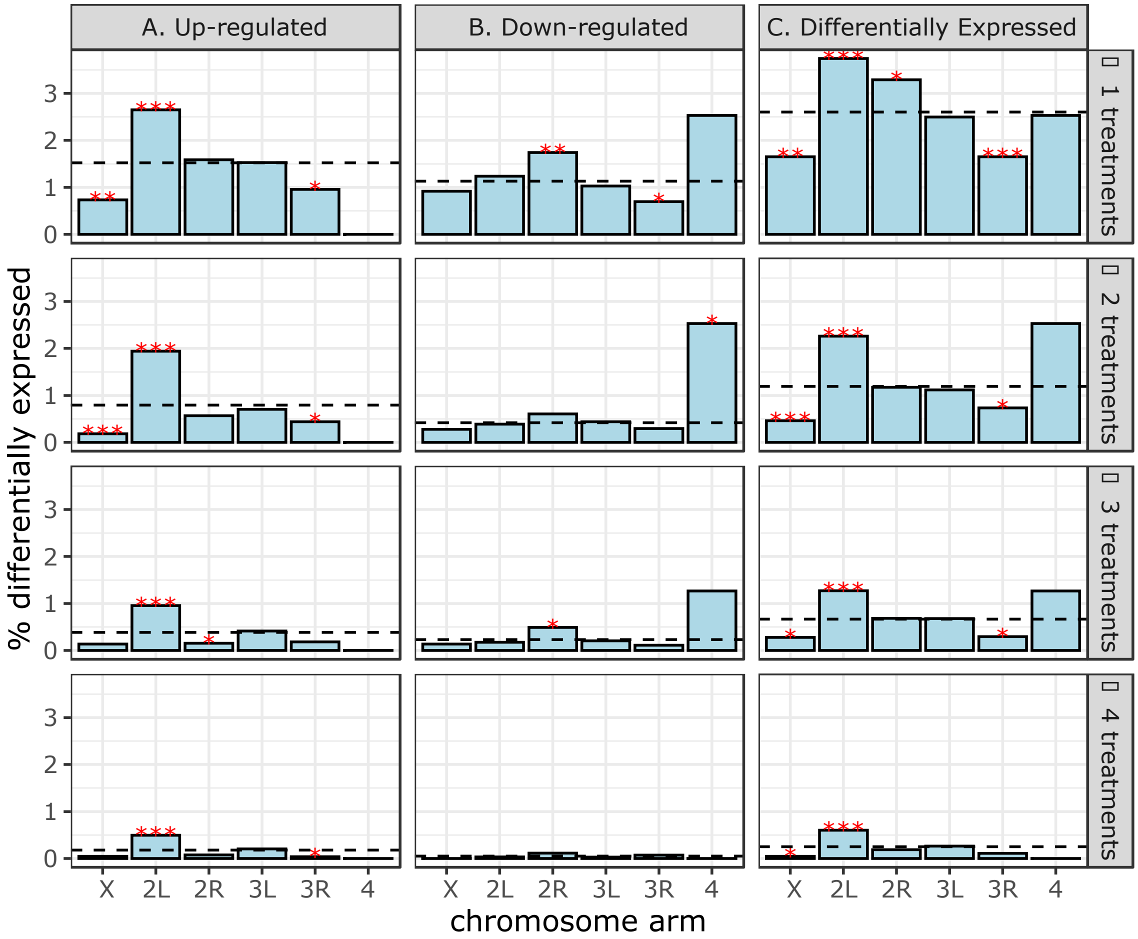

Supplemental Figure S2. The percent of genes on each chromosome arm that are differentially expressed following infection with at least 1, 2, 3, or 4 different bacteria in male *D. melanogaster* is shown. Genes are either (A) up-regulated, (B) downregulated, or (C) differentially expressed (sum of up- and down-regulated). The percent of differentially expressed genes across the entire genome is shown as a dashed line. Asterisks indicate chromosomes where the percent of genes is significantly different from the rest of the genome (**P*<0.05, ***P*<0.005, or ****P*<0.0005 in Fisher's exact test).

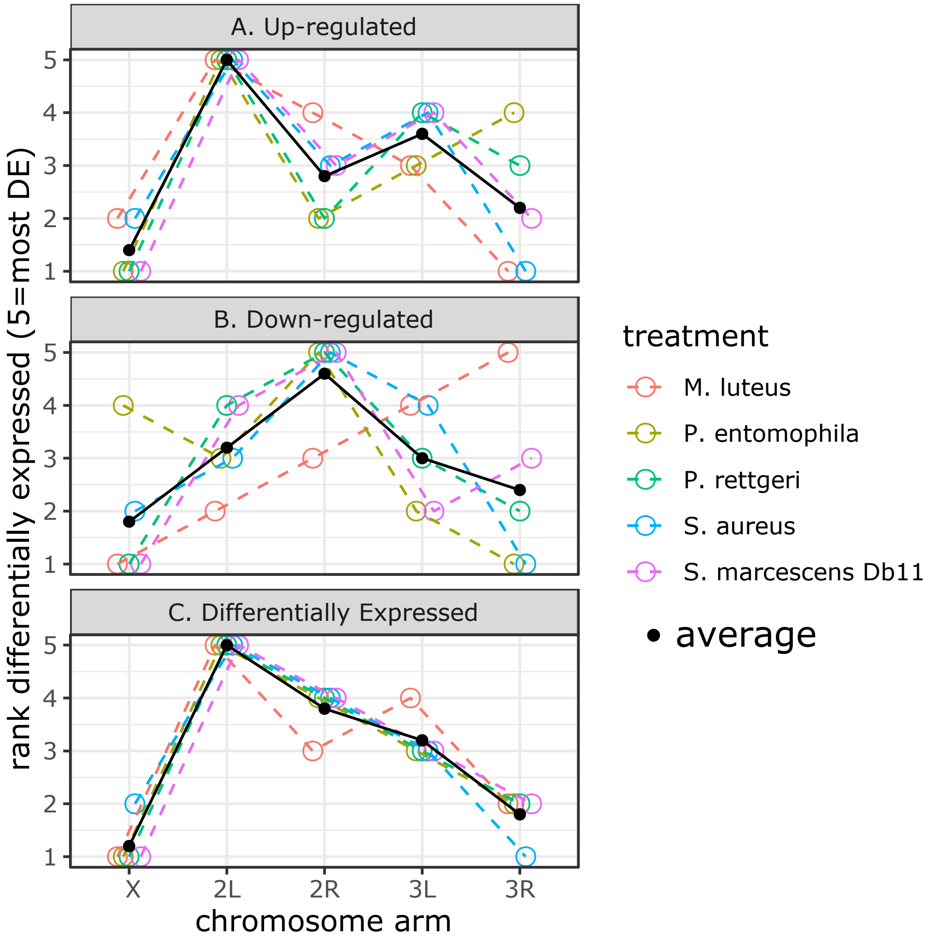

Supplemental Figure S3. The rank order of chromosome arms is shown according to the percent of differentially expressed genes in each of five different bacterial treatments. The chromosome arm that has the highest percent of differentially expressed genes is ranked as 5, and the chromosome with the lowest percent is ranked as 1. Genes are either (A) up-regulated, (B) downregulated, or (C) differentially expressed (sum of up- and down-regulated). The mean ranks for each chromosome are shown as black dots and solid lines.

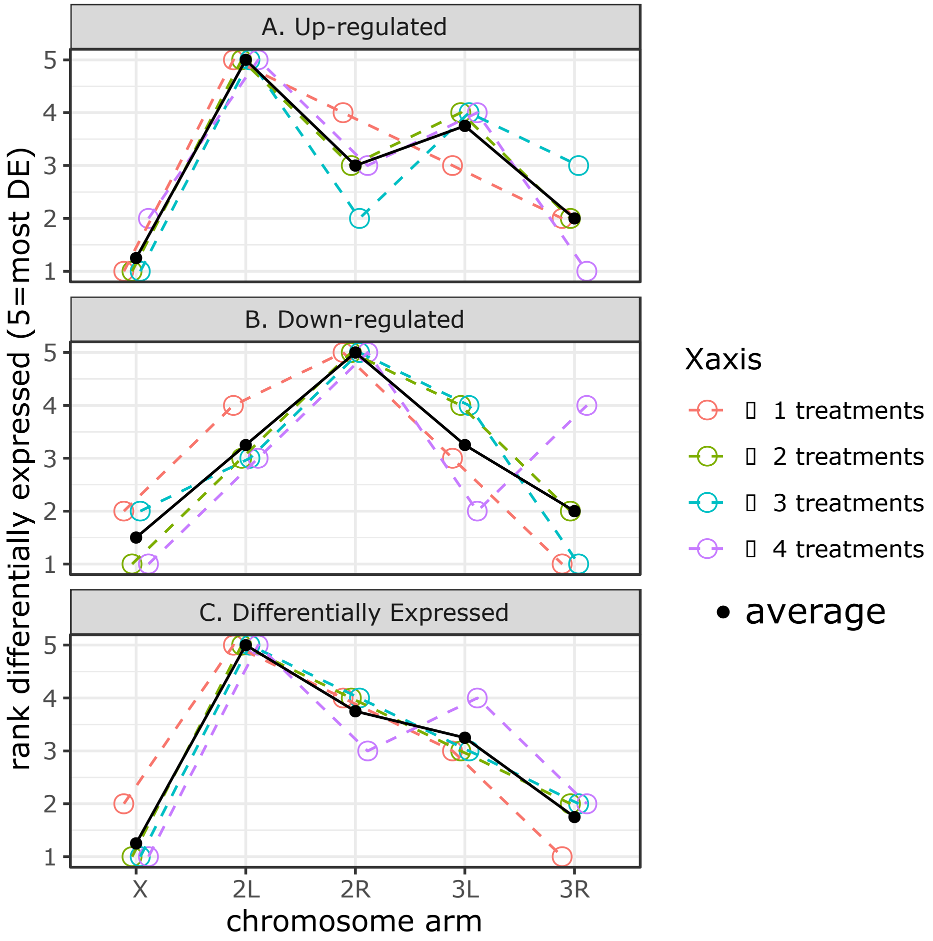

Supplemental Figure S4. The rank order of chromosome arms is shown according to the percent of differentially expressed genes in at least one, two, three, or four bacterial treatments. The chromosome arm that has the highest percent of differentially expressed genes is ranked as 5, and the chromosome with the lowest percent is ranked as 1. Genes are either (A) up-regulated, (B) downregulated, or (C) differentially expressed (sum of up- and down-regulated). The mean ranks for each chromosome are shown as black dots and solid lines.

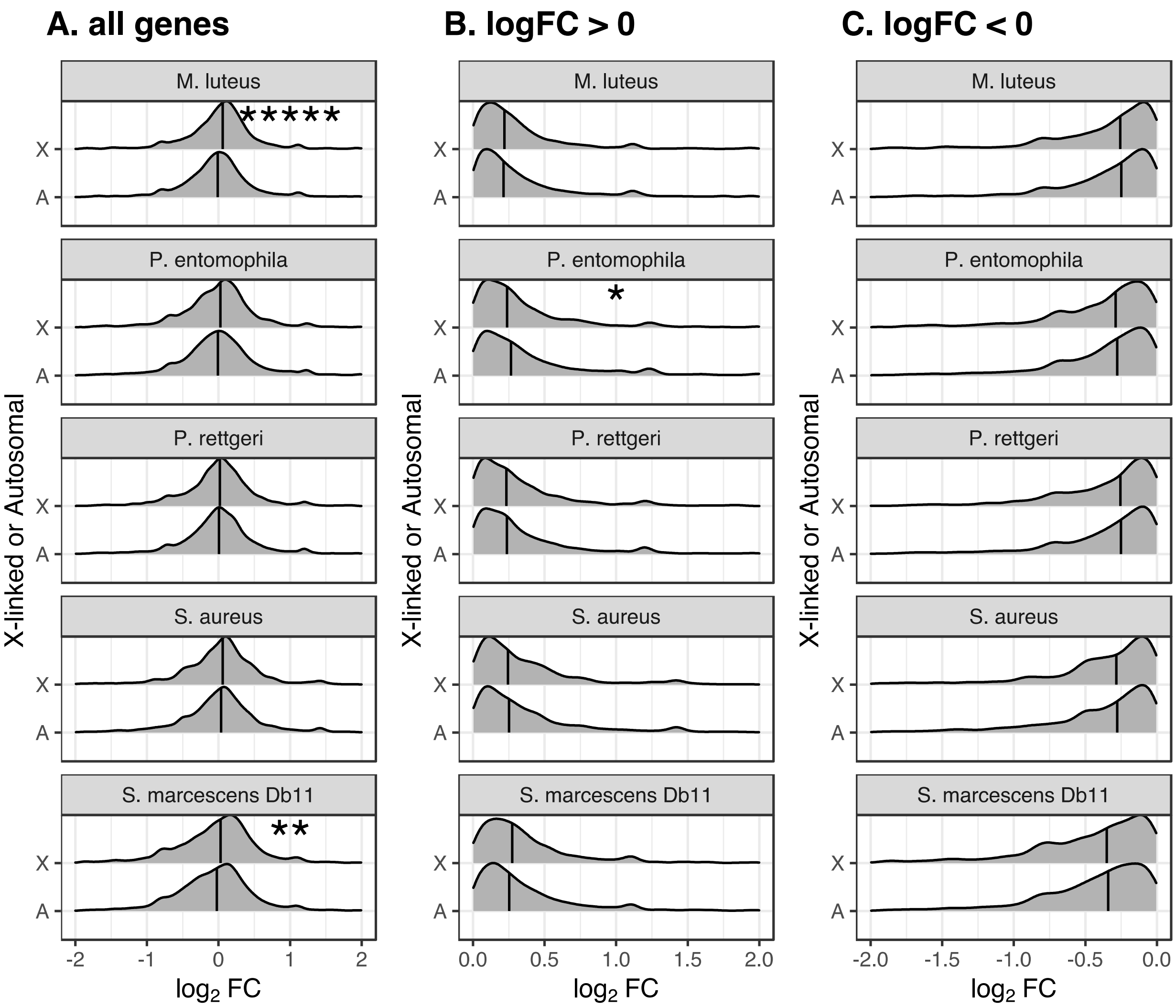

Supplemental Figure S5. Distributions of log_2FC following bacterial infection for X-linked (X) and autosome (A) genes in *D. melanogaster* males. Asterisks show significant differences between X and autosomes within a treatment (*P<0.05; **P<0.005; *****P<0.000005; Mann-Whitney test). Data are shown for (A) all genes, (B) genes with $log_2FC>0$, and (C) genes with $log_2FC<0$.

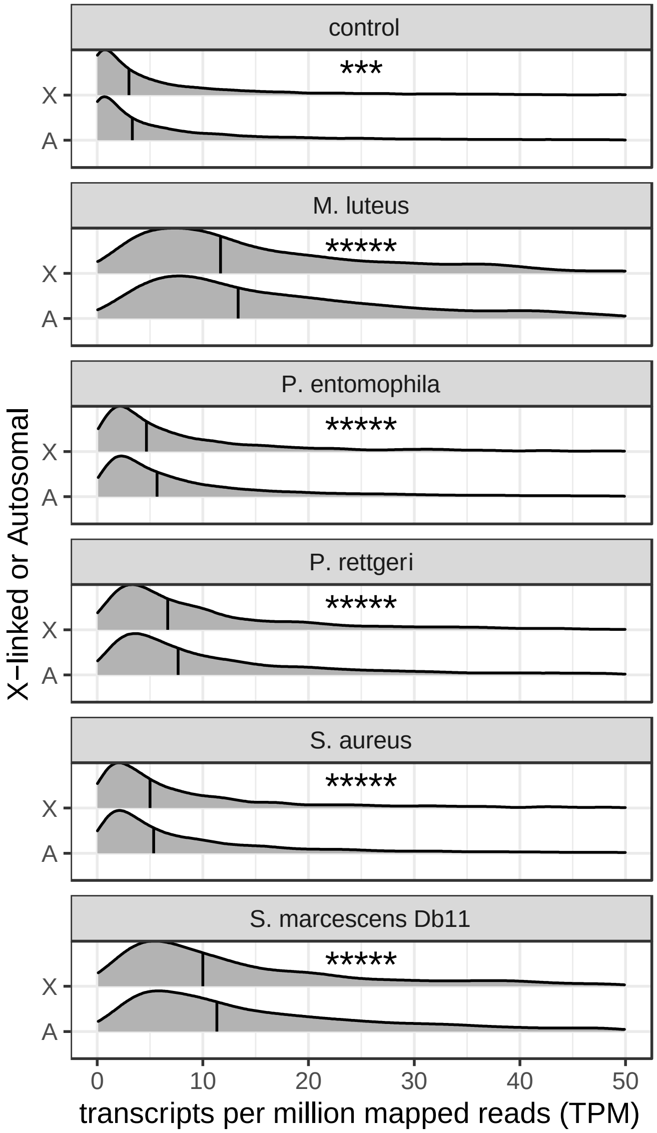

Supplemental Figure S6. Distributions of transcripts per million mapped reads (TPM) in the control or following bacterial infection for X-linked (X) and autosome (A) genes in *D. melanogaster* males. Asterisks show significant differences between X and autosomes within control or treatment (***P<0.0005; *****P<0.000005; Mann-Whitney test).

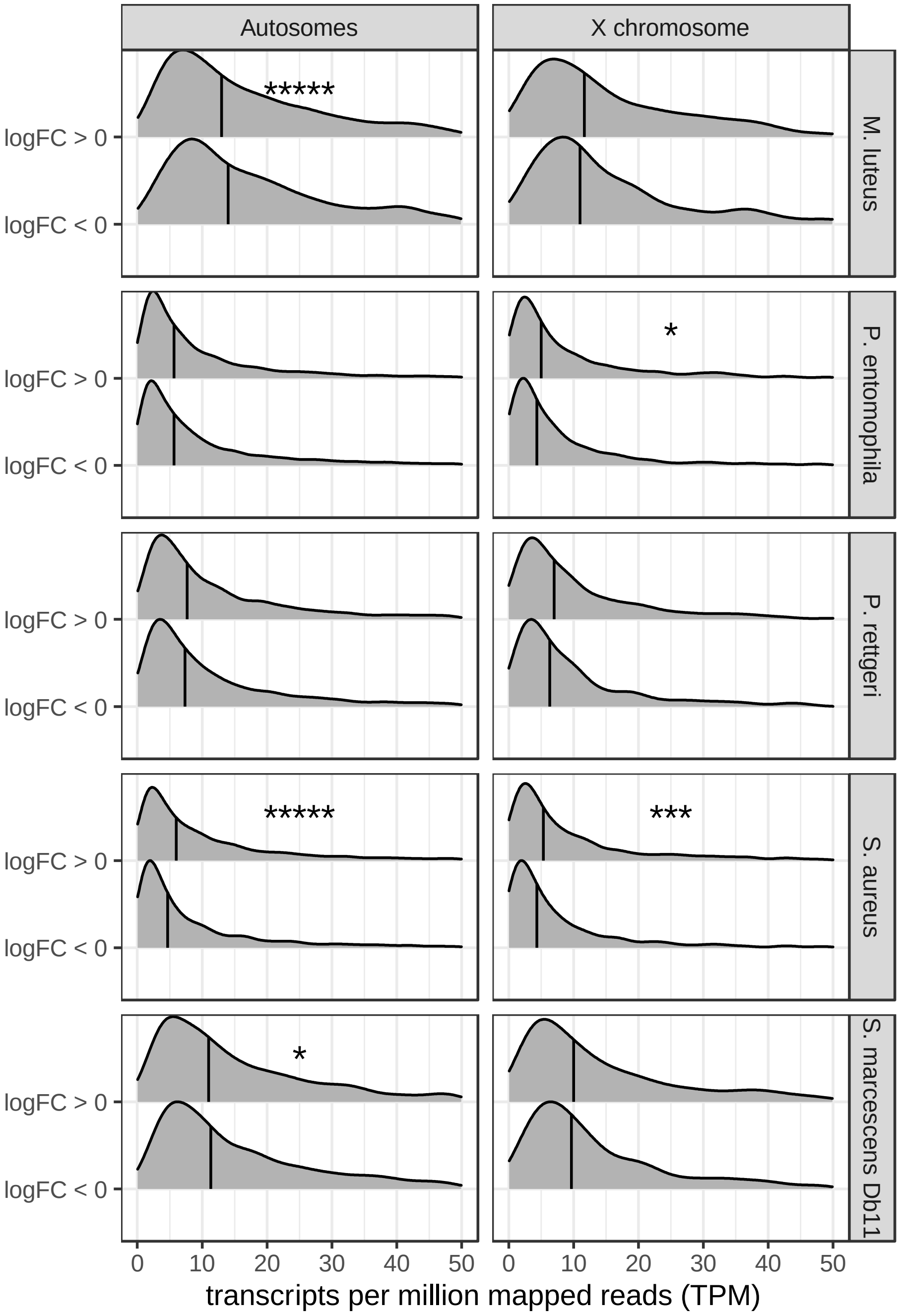

Supplemental Figure S7. Distributions of transcripts per million mapped reads (TPM) following bacterial infection for autosomal and X chromosome genes in *D. melanogaster* males. Genes were divided into those with logFC>0 and those with logFC<0. Asterisks show significant differences in TPM between logFC>0 and logFC<0 within a treatment (*P<0.05; ***P<0.0005; *****P<0.000005; Mann-Whitney test).

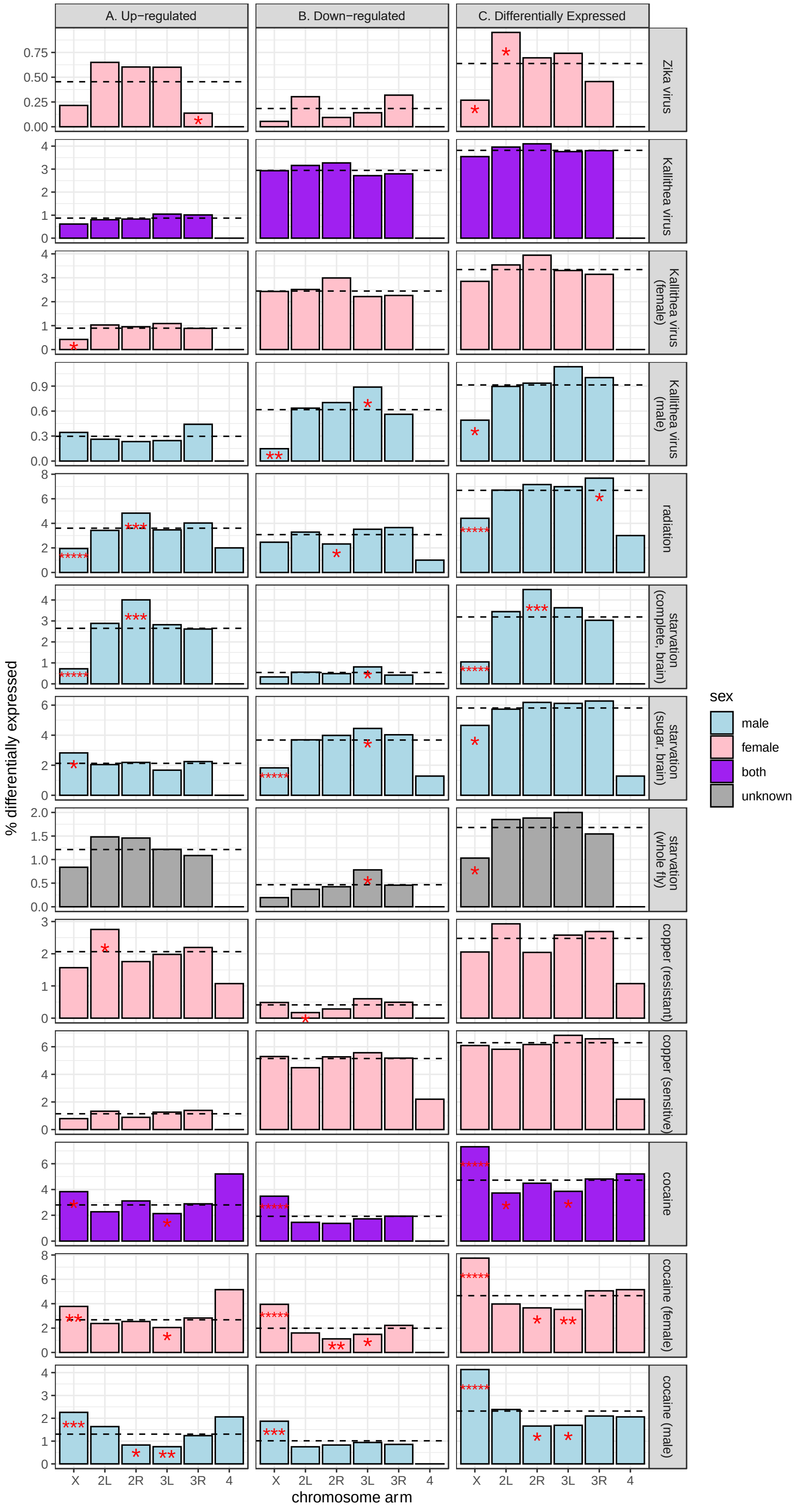

A. Up−regulated
B. Down−regulated
C. Differentially Expressed
Zika virus
Kallithea virus
Kallithea virus (female)
Kallithea virus (male)
radiation
starvation (complete, brain)
starvation (sugar, brain)
starvation (whole fly)
copper (resistant)
copper (sensitive)
cocaine
cocaine (female)
cocaine (male)
% differentially expressed
chromosome arm
X
2L
2R
3L
3R
4
sex
male
female
both
unknown

Supplemental Figure S8. The percent of genes on each chromosome arm that are differentially expressed following viral and abiotic treatments is shown. Bars are colored by the sex of the flies used in each treatment (see legend). Genes are either (A) up-regulated, (B) downregulated, or (C) differentially expressed (sum of up- and down-regulated). The percent of differentially expressed genes across the entire genome is shown as a dashed line. Asterisks indicate chromosomes where the percent of genes is significantly different from the rest of the genome (*$P<0.05$, **$P<0.005$, or ***$P<0.0005$ in Fisher's exact test).

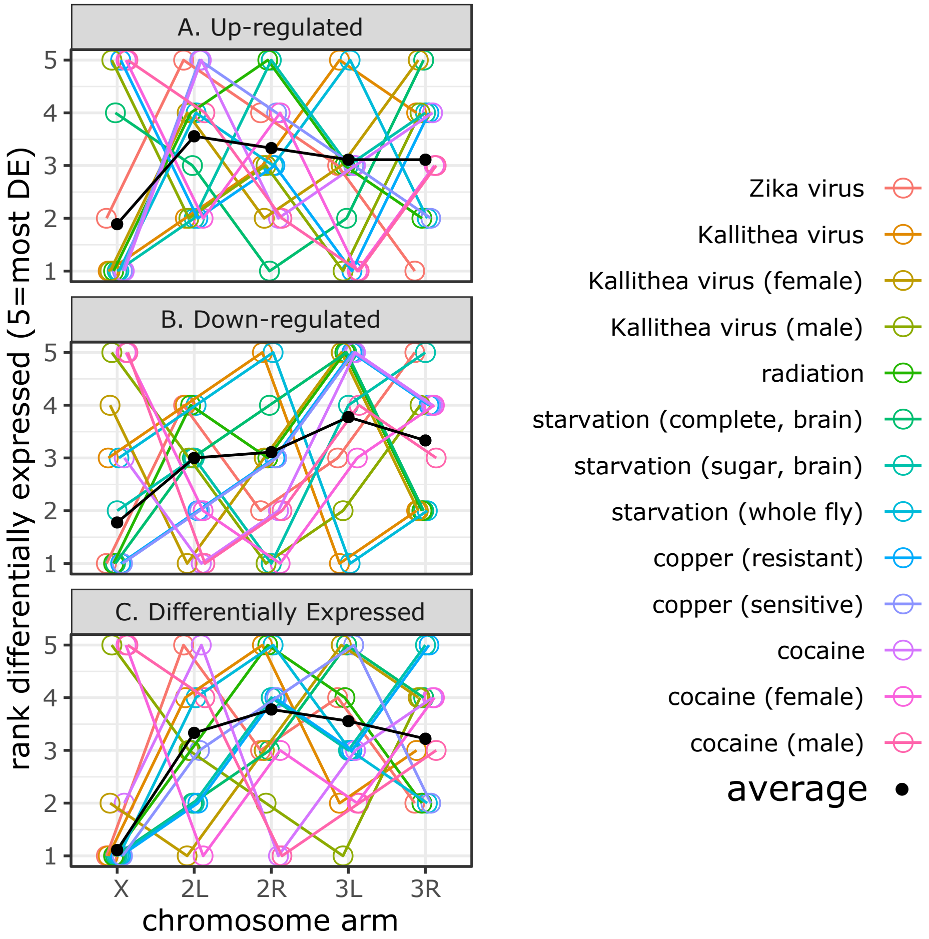

Supplemental Figure S9. The rank order of chromosome arms is shown according to the percent of differentially expressed genes in viral and abiotic treatments. The chromosome arm that has the highest percent of differentially expressed genes is ranked as 5, and the chromosome with the lowest percent is ranked as 1. Genes are either (A) up-regulated, (B) downregulated, or (C) differentially expressed (sum of up- and down-regulated). The mean ranks for each chromosome are shown as black dots and solid lines.

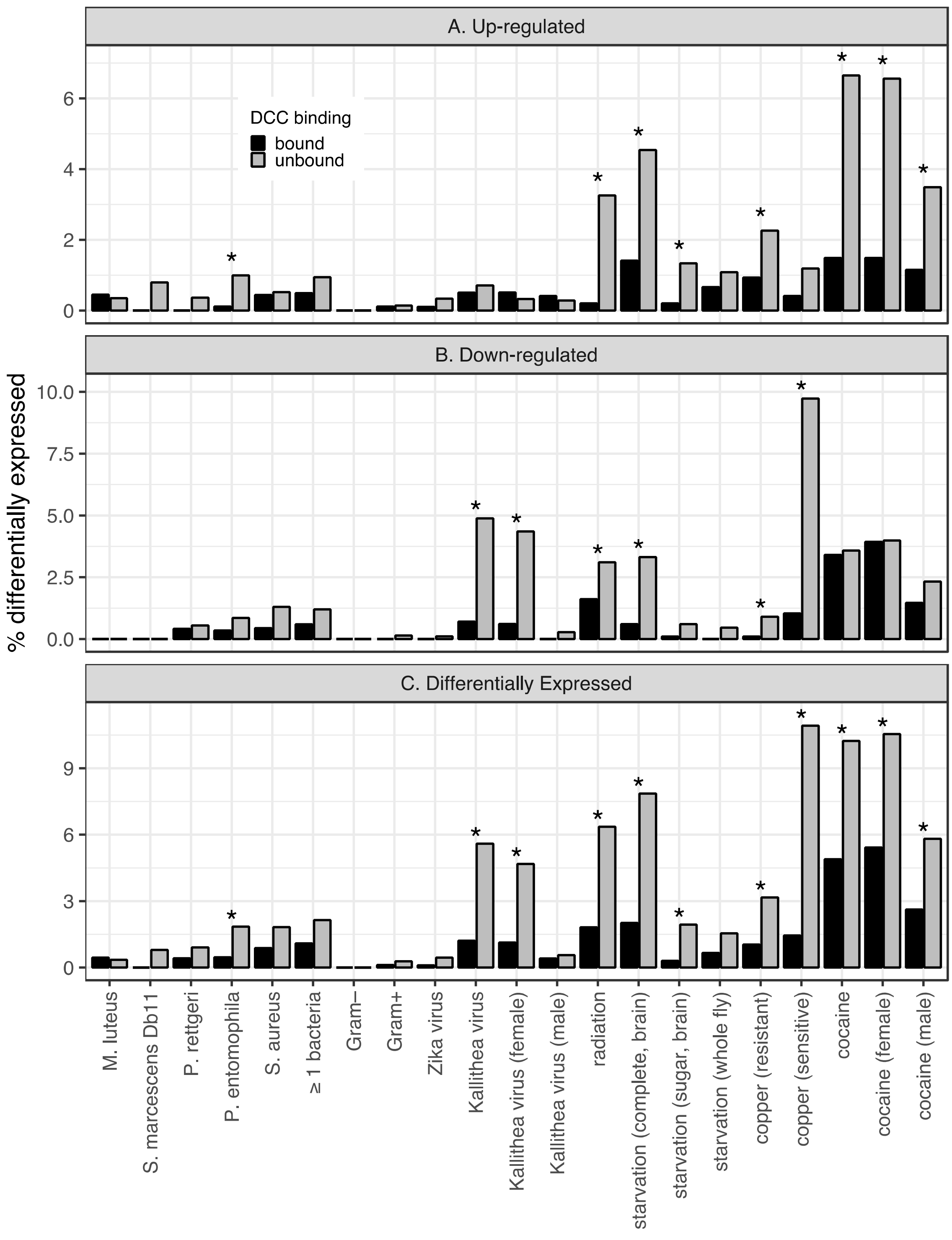

Supplemental Figure S10. The percent of X-linked DCC-bound (black) and unbound (gray) genes that are up-regulated (A), down-regulated (B), or differentially expressed (C) are shown for each treatment and sample type (in parentheses). Asterisks show a significant difference in the percent between DCC-bound and unbound genes (*P<0.05 in Fisher's exact test).

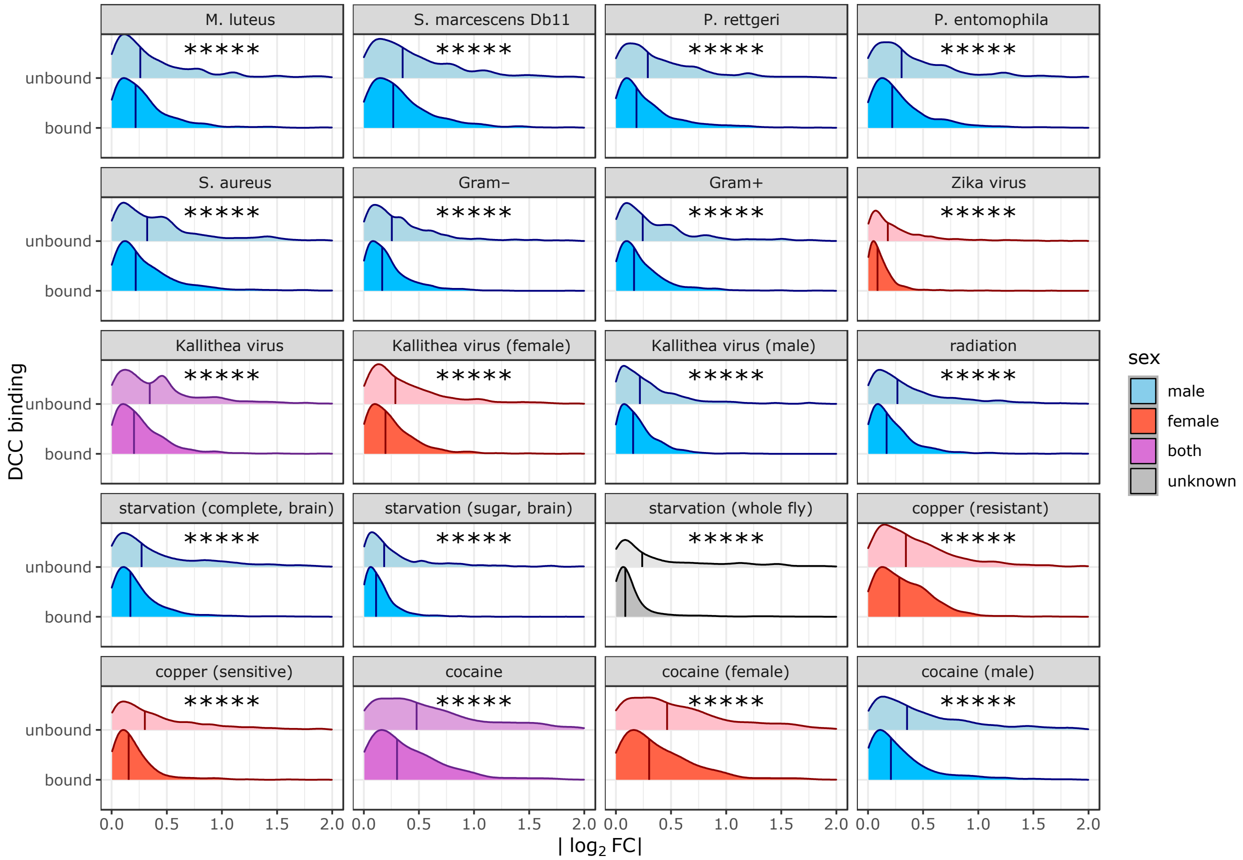

Supplemental Figure S11. Distributions of |log$_2$FC| following biotic and abiotic treatments for X-linked genes that are either bound by the DCC or unbound. Colors indicate the sex of the flies used in the experiments. Asterisks show significant differences in |log$_2$FC| between DCC bound and unbound genes within a treatment (*****P<0.000005; Mann-Whitney test).

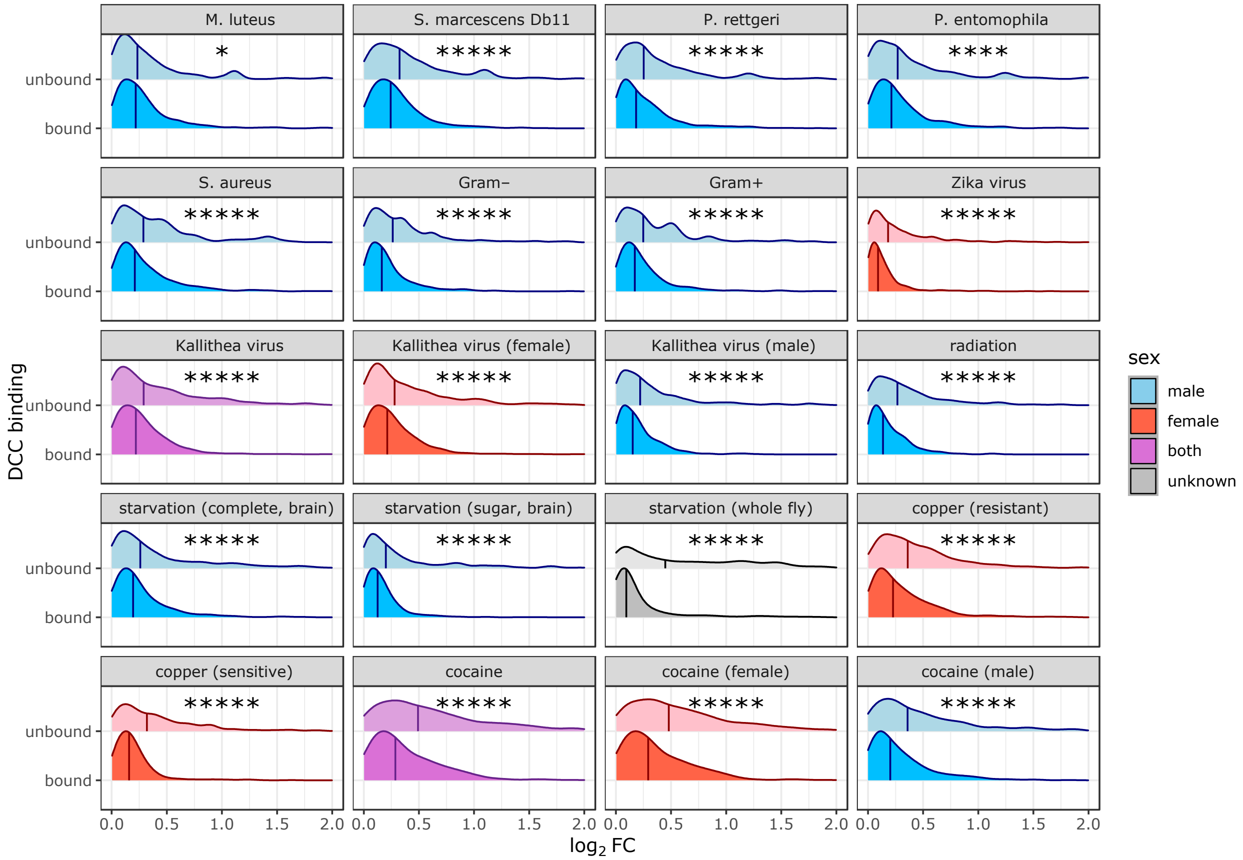

Supplemental Figure S12. Distributions of $\log_2$FC following biotic and abiotic treatments for X-linked genes that are either bound by the DCC or unbound. Only genes with $\log_2$FC>0 are plotted. Colors indicate the sex of the flies used in the experiments. Asterisks show significant differences in $\log_2$FC between DCC bound and unbound genes within a treatment (*P<0.05; ****P<0.00005; *****P<0.000005; Mann-Whitney test).

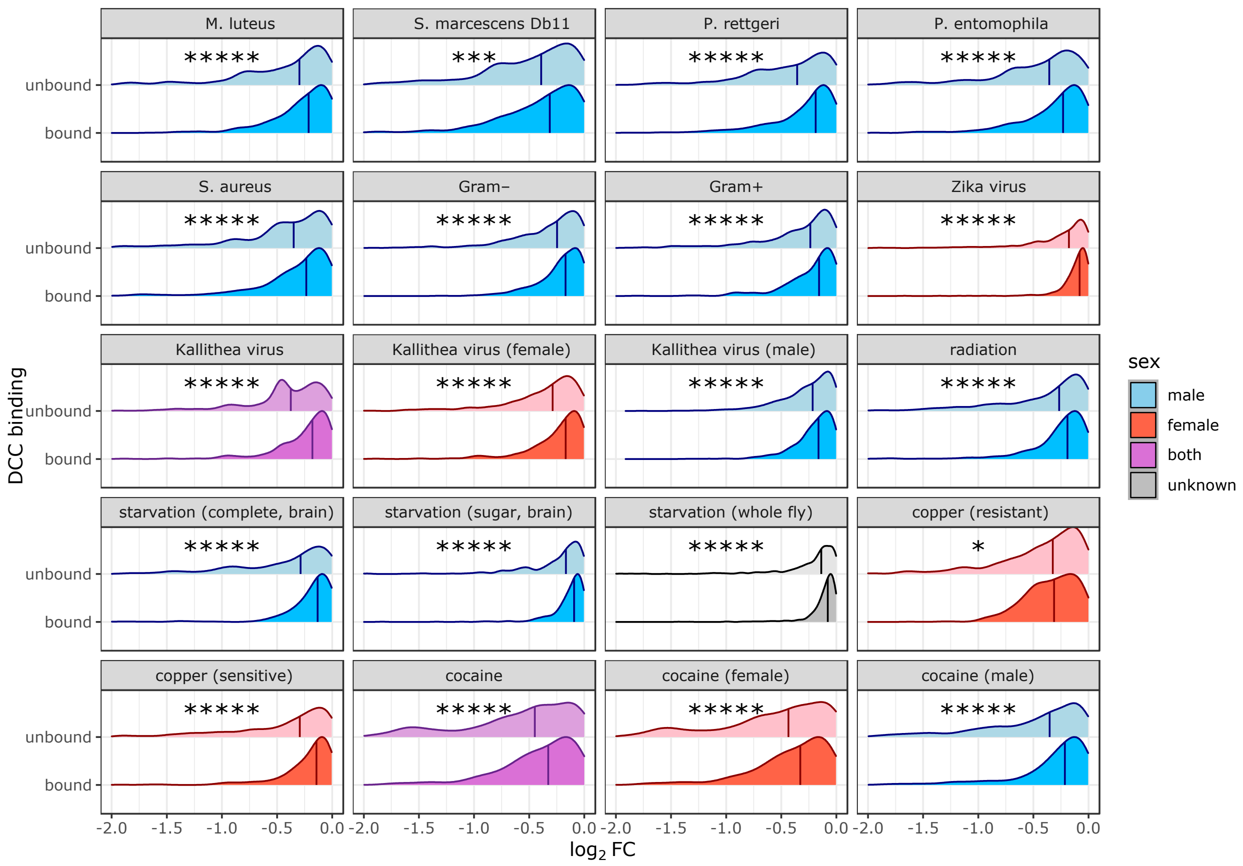

Supplemental Figure S13. Distributions of $\log_2$FC following biotic and abiotic treatments for X-linked genes that are either bound by the DCC or unbound. Only genes with $\log_2$FC<0 are plotted. Colors indicate the sex of the flies used in the experiments. Asterisks show significant differences in $\log_2$FC between DCC bound and unbound genes within a treatment (*P<0.05; ***P<0.0005; *****P<0.000005; Mann-Whitney test).

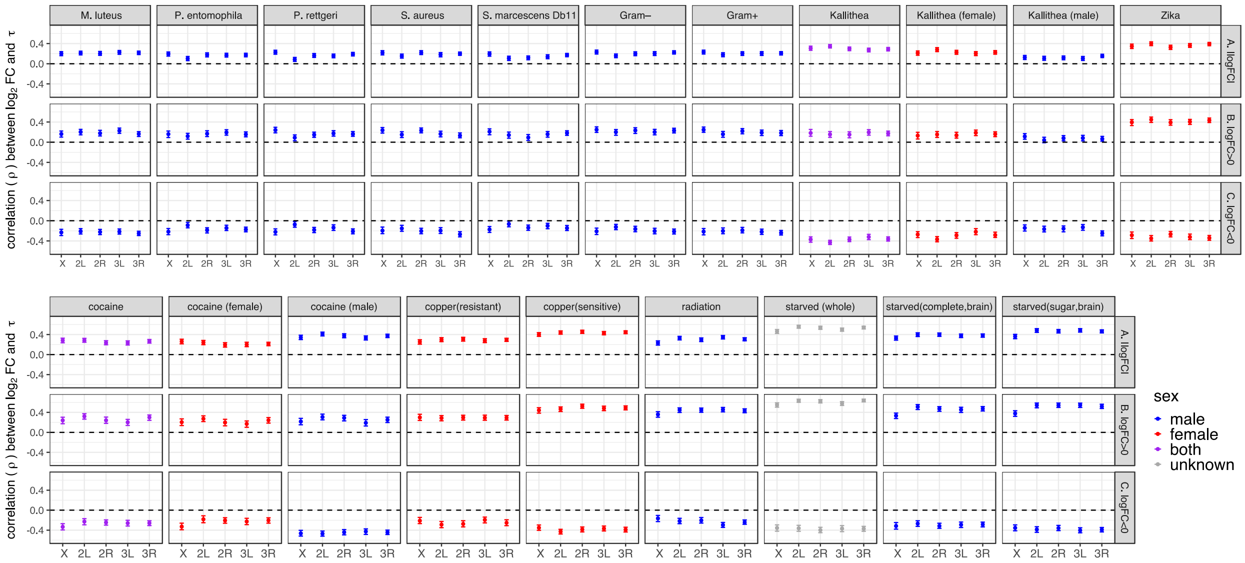

Supplemental Figure S14. Spearman's rank order correlations (ρ) between $\log_2$FC and tissue-specificity (τ) are shown for each chromosome in each treatment. Error bars show the 95% confidence interval from 1,000 bootstrap replicate samples of genes on each chromosome. The analysis was performed on absolute values of $\log_2$FC of all genes (A), genes with positive $\log_2$FC>0 (B), or genes with negative $\log_2$FC<0 (C). Tissue-specificity values (τ) were calculated using all 14 adult tissues.

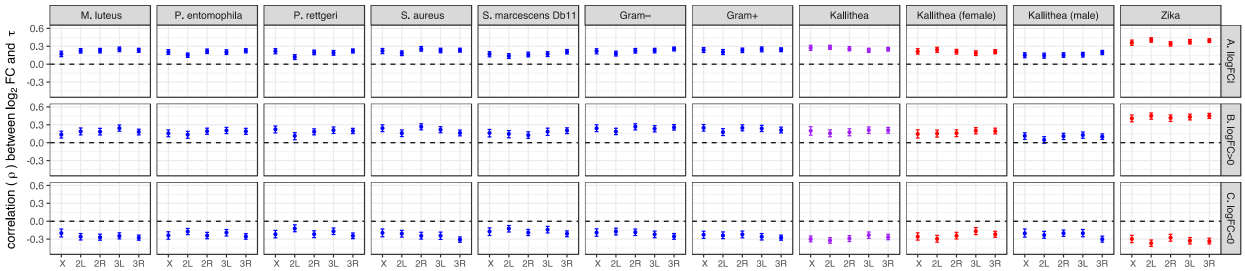

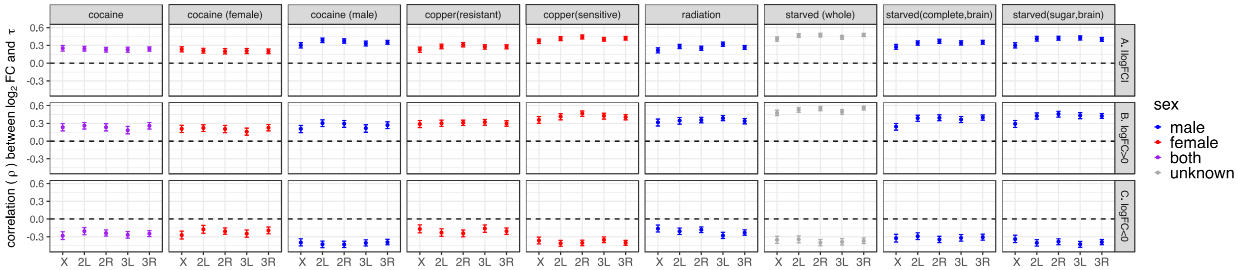

Supplemental Figure S15. Spearman's rank order correlations (ρ) between $\log_2$FC and tissue-specificity (τ) are shown for each chromosome in each treatment. Error bars show the 95% confidence interval from 1,000 bootstrap replicate samples of genes on each chromosome. The analysis was performed on absolute values of $\log_2$FC of all genes (A), genes with positive $\log_2$FC>0 (B), or genes with negative $\log_2$FC<0 (C). Tissue-specificity values (τ) were calculated using 10 non-sex-specific tissues.

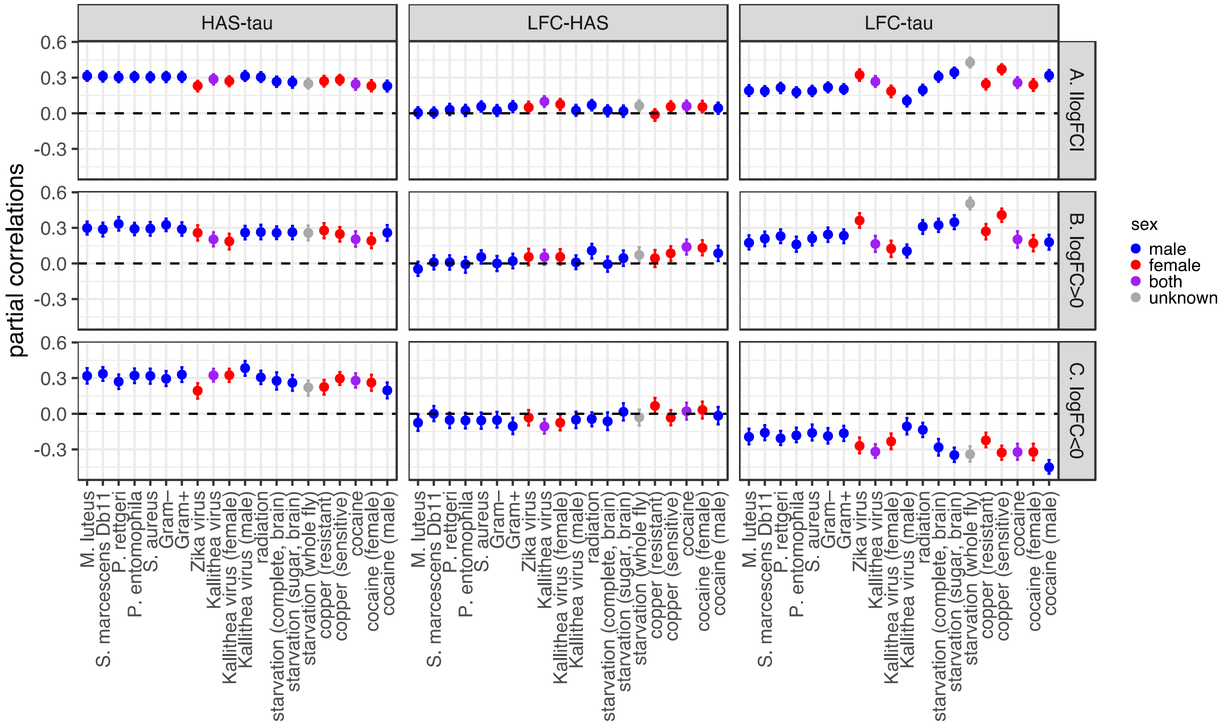

Supplemental Figure S16. Partial correlations between distance from a DCC high affinity site (HAS), tissue-specificity (tau), and log$_2$FC (LFC) are shown for each treatment. Error bars show the 95% confidence interval from 1,000 bootstrap replicate samples of X-linked genes. The analysis was performed on absolute values of log$_2$FC of all genes (A), genes with positive log$_2$FC>0 (B), or genes with negative log$_2$FC<0 (C). Tissue-specificity values (τ) were calculated using all 14 adult tissues. HAS were taken from Alekseyenko *et al*. (2008).

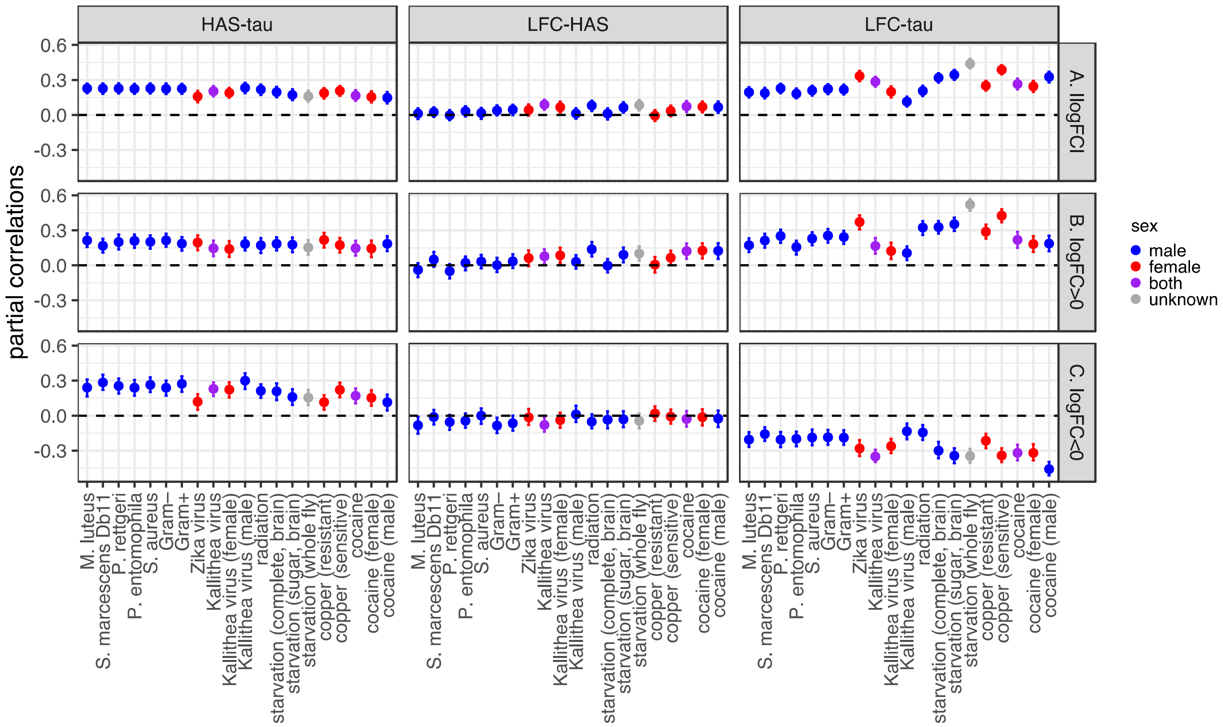

Supplemental Figure S17. Partial correlations between distance from a DCC high affinity site (HAS), tissue-specificity (tau), and log_2FC (LFC) are shown for each treatment. Error bars show the 95% confidence interval from 1,000 bootstrap replicate samples of X-linked genes. The analysis was performed on absolute values of log_2FC of all genes (A), genes with positive $log_2FC>0$ (B), or genes with negative $log_2FC<0$ (C). Tissue-specificity values (τ) were calculated using all 14 adult tissues. HAS were taken from Straub *et al*. (2008).

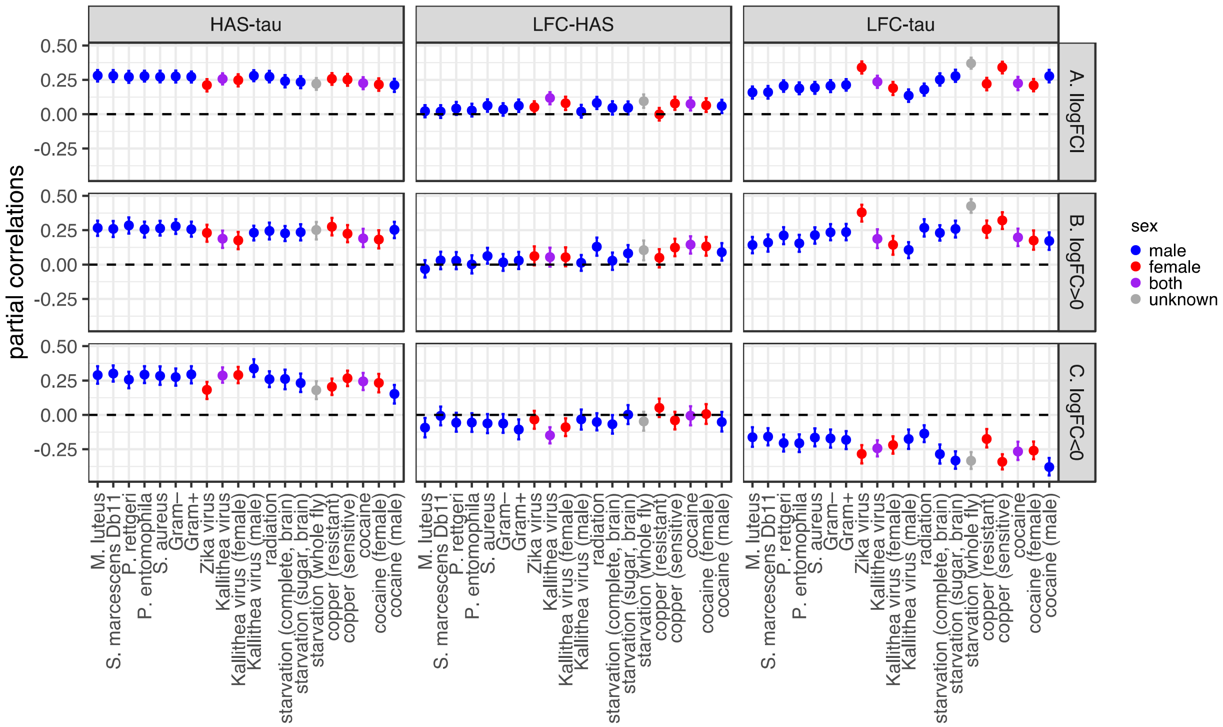

Supplemental Figure S18. Partial correlations between distance from a DCC high affinity site (HAS), tissue-specificity (tau), and $\log_2$FC (LFC) are shown for each treatment. Error bars show the 95% confidence interval from 1,000 bootstrap replicate samples of X-linked genes. The analysis was performed on absolute values of $\log_2$FC of all genes (A), genes with positive $\log_2FC>0$ (B), or genes with negative $\log_2FC<0$ (C). Tissue-specificity values (τ) were calculated using 10 non-sex-specific tissues. HAS were taken from Alekseyenko et al. (2008).

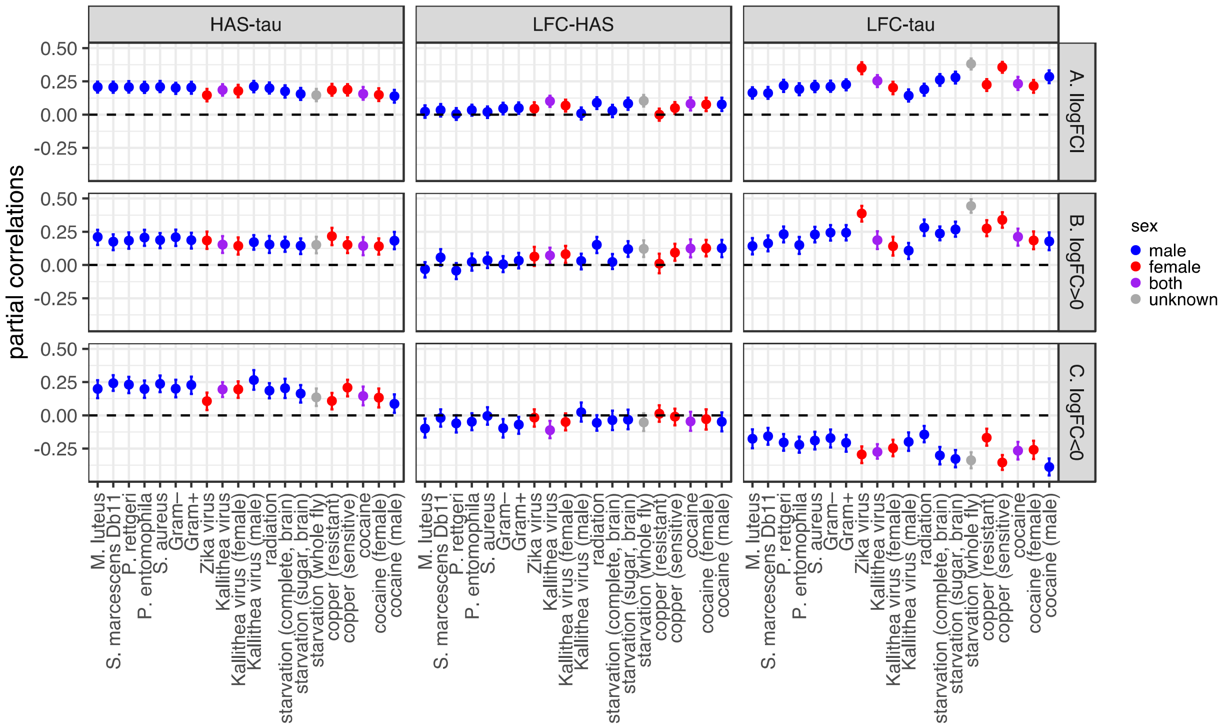

Supplemental Figure S19. Partial correlations between distance from a DCC high affinity site (HAS), tissue-specificity (tau), and $\log_2$FC (LFC) are shown for each treatment. Error bars show the 95% confidence interval from 1,000 bootstrap replicate samples of X-linked genes. The analysis was performed on absolute values of $\log_2$FC of all genes (A), genes with positive $\log_2$FC>0 (B), or genes with negative $\log_2$FC<0 (C). Tissue-specificity values (τ) were calculated using 10 non-sex-specific-tissues. HAS were taken from Straub *et al*. (2008).

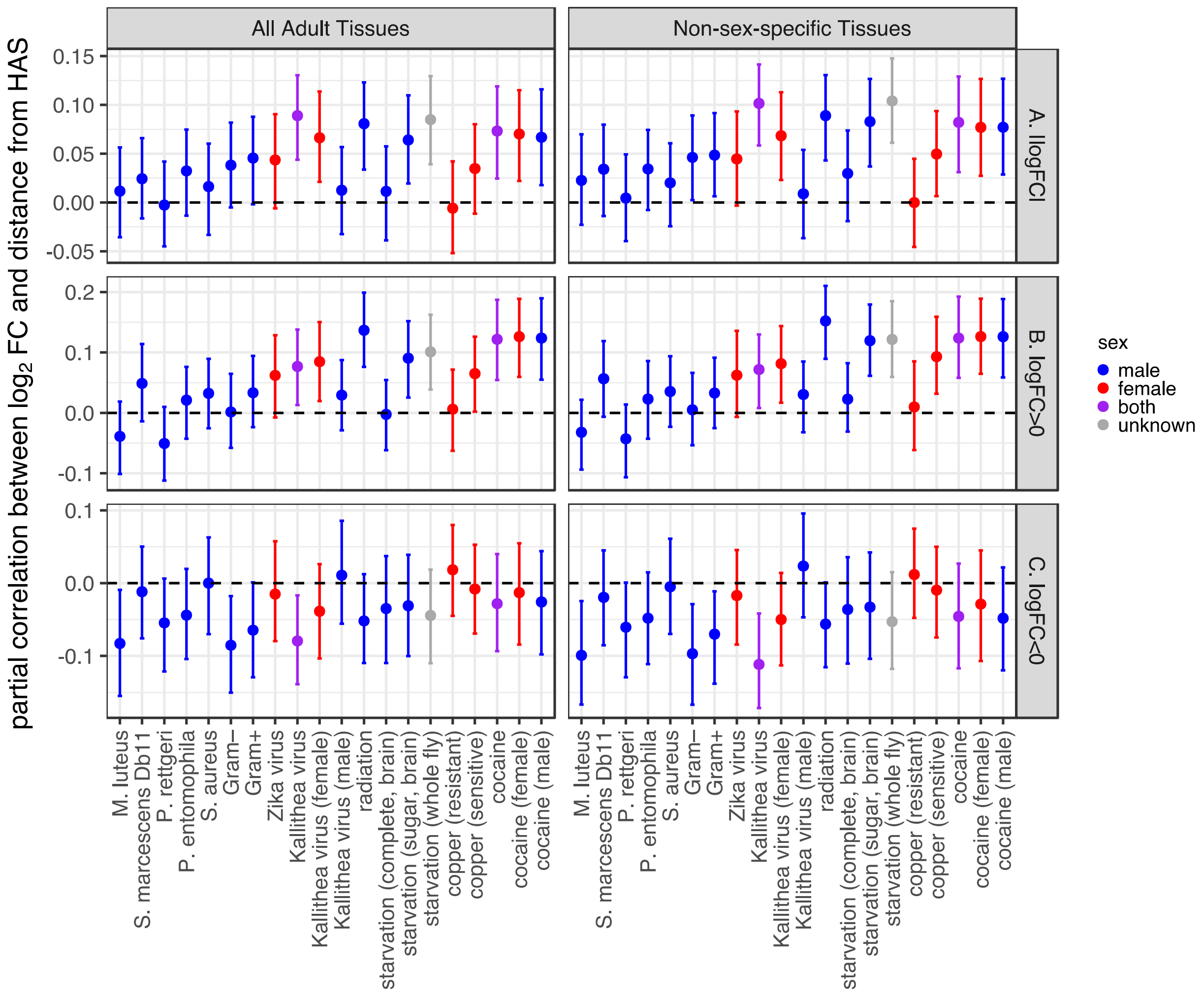

Supplemental Figure S20. Partial correlations between $\log_2$ fold-change in expression between treatment and control ($\log_2$FC) and distance from a dosage compensation complex high affinity site (HAS). Partial correlations were calculated based on rank order correlations between $\log_2$FC, distance from an HAS, and tissue expression breadth. Each dot shows the partial correlation between $\log_2$FC and distance from an HAS, with the error bars representing 95% confidence intervals from 1,000 bootstrap replicates of the data. The X-axis shows the specific treatment. Dots and error bars are colored based on the sex of the flies used in the experiment (see legend). HAS were obtained from the Straub *et al.* (2008) data set. Expression breadth was calculated using microarray data from either 14 unique adult tissues (left) or 10 adult tissues that are not sex-specific (right). Partial correlations are plotted with $|\log_2\text{FC}|$ values for all genes (A), only genes with $\log_2\text{FC}>0$ (B), and only genes with $\log_2\text{FC}<0$ (C).
